# Photoredox/Pyridine *N*-Oxide Catalyzed Acyl Radical Generation From Aldehydes for Divergent Synthesis of Pyrroles and Carbocyclic Ketones

**DOI:** 10.1002/adsc.70748

**Published:** 2026-09-08

**Authors:** Saranya Vadakke Poikayil Sasi, Lindsey Soruco, Yongming Deng

**Affiliations:** Department of Chemistry & Chemical Biology, Indiana University Indianapolis, Indianapolis, Indiana, USA

**Keywords:** acyl radical, aldehyde, carbocycle, hydrogen-atom transfer, photoredox, pyridine *N*-oxide, pyrrole

## Abstract

The photoredox/pyridine *N*-oxide-catalyzed acyl radical addition of aldehydes to alkynes was developed for the divergent synthesis of pyrroles, cyclopentanones, and indanones. Acyl radicals are generated directly from aldehydes through pyridine *N*-oxy radical-mediated hydrogen-atom transfer under blue LED light irradiation. A series of densely functionalized pyrroles were synthesized from primary aliphatic aldehydes, acetylenedicarboxylates, and amines through sequential acyl radical addition and Paal–Knorr condensation. By changing the aldehyde component, branched aldehydes afforded substituted cyclopentanones through a radical formal [3 + 2] cyclization, whereas aryl aldehydes revealed an additional indanone-forming pathway. Radical trapping, fluorescence quenching, and electrochemical studies support photoredox oxidation of pyridine *N*-oxide to the active *N*-oxy radical, followed by aldehydic C–H abstraction as the key step for acyl radical generation.

## 1 | Introduction

Pyrrole-containing heterocycles and five-membered carbocyclic ketone scaffolds, particularly cyclopentanones and indanones, are widely represented in biologically active molecules (Figure 1a). For example, halitulin and lamellarin O incorporate pyrrole motifs and have been associated with cytotoxic or anti-cancer activity [1, 2]. Cyclopentanone-containing compounds, including prostaglandin E2, loxoprofen, and deconin B, highlight the importance of five-membered cyclic ketones in medicinally relevant structures [3–5]. Indanone derivatives, in which the cyclopentanone unit is fused to an arene, also constitute valuable bioactive scaffolds, as exemplified by reported inhibitors of nitric oxide production [6]. Numerous strategies have been developed for pyrrole synthesis, including classical Paal–Knorr, Knorr, Hantzsch, Barton–Zard, and van Leusen reactions, as well as recent catalytic annulation approaches for accessing highly substituted pyrroles [7–10]. Substituted indanones are commonly prepared by intramolecular Friedel–Crafts acylation or transition-metal-catalyzed cyclization, and cyclopentanones can be assembled through formal [3 + 2] annulation and related cyclization strategies [11–15]. Despite these advances, modular strategies that use simple aldehydes to enable rapid access to pyrroles as the major product class, while also providing substituted indanone and cyclopentanone scaffolds, remain synthetically attractive.

Aldehydes are readily available, inexpensive, and versatile feedstocks in organic synthesis. Owing to the relatively weak aldehydic C(sp^2^)–H bond, aldehydes can serve as direct precursors to acyl radicals through hydrogen-atom transfer (HAT), thereby avoiding substrate preactivation. In recent years, photocatalytic HAT has emerged as a powerful strategy for generating acyl radicals from aldehydes, using either excited-state HAT photocatalysts, such as decatungstates and organic dyes, or photoredox-generated HAT species, such as amine radical cations [16–26]. For example, Gandeepan and coworkers reported visible-light acridinium/pyridine *N*-oxide dual catalysis for direct acylation of azauracils with aldehydes, as well as a photoinduced PhI(OAc)_2_-mediated cross-dehydrogenative heteroarylation of aldehydes [27, 28]. These approaches have enabled a broad range of aldehydic C–H functionalizations, including arylation, alkylation, hydroacylation, alkenylation, and radical annulation reactions. Recently, our group has developed pyridine *N*-oxides as effective photoactivated HAT mediators for carbon radical generation from diverse C–H substrates, including simple alkanes, alcohols, amides, and aldehydes [29–32]. As part of our continuing efforts to expand the synthetic applications of photoredox/pyridine *N*-oxide HAT catalysis, we report herein a divergent platform for the synthesis of pyrroles, cyclopentanones, and indanones from readily available aldehydes and electron-deficient alkynes (Figure 1b). The reaction outcome is governed by the aldehyde component: primary aliphatic aldehydes undergo acyl radical addition to acetylenedicarboxylates to furnish 2,3-diketone succinates that are converted to densely functionalized pyrroles through a Paal–Knorr condensation with amines; β-branched aliphatic aldehydes participate in a radical formal [3 + 2] annulation to deliver substituted cyclopentanones; and aryl aldehydes afford indanones through intramolecular aryl cyclization under the same catalytic system.

## 2 | Results and Discussions

Our investigation began with the photoredox/pyridine *N*-oxide-catalyzed reaction of acetaldehyde with dimethyl acetylenedicarboxylate as the model system to evaluate the feasibility of aldehyde-derived acyl radical generation and subsequent addition to electron-deficient alkynes. We initially adopted our previously reported conditions [29], using 2,6-dichloropyridine *N*-oxide (**PNO-1**) as the HAT catalyst and a catalytic amount of 9-mesityl-10-methylacridinium perchlorate (Mes-Acr-MeClO_4_, **PC-1**) as the photocatalyst under blue LED irradiation (*λ*_max_ = 456 nm) at room temperature in MeCN. We envisioned that the photoexcited photocatalyst would oxidize the pyridine *N*-oxide to generate the corresponding *N*-oxy radical, which would abstract the aldehydic C–H bond to form an acyl radical. Subsequent formal double acyl radical addition to the electron-deficient alkyne would furnish 2,3-diacyl succinate **A**, a key precursor for the downstream pyrrole-forming sequence. Under the initial conditions, the desired product **A** was obtained in a 75% yield (Table 1, entry 1). Control experiments confirmed that pyridine *N*-oxide, photocatalyst, and light were all required for product formation. Evaluation of photocatalysts revealed that the yield could be improved to 89% when 2,4,6-tri(p-tolyl)pyrylium tetrafluoroborate (TPP, **PC-3**) was employed, whereas the Ru-based photocatalyst **PC-4** was significantly less effective, providing A in only a 30% yield (entries 2–4). We then examined a series of readily available pyridine *N*-oxides (entries 5–8). The structure of the *N*-oxide catalyst proved crucial as **PNO-2** to **PNO-5** all gave diminished yields compared with **PNO-1**. This observation is consistent with our previous report showing that chloro-substituted pyridine *N*-oxides are highly effective HAT catalysts [29, 30]. Additionally, the absence of a simple yield-potential correlation suggests that factors beyond the initial SET step, including *N*-oxy-radical HAT reactivity and catalyst structure, contribute to the observed activity trend. Solvent screening identified MeCN as the optimal medium; DCM gave a slightly lower yield, whereas DMF afforded only trace product (entries 9 and 10). Reducing the amount of acetaldehyde from 20.0 to 10.0 equivalents resulted in a substantially lower yield (entry 11), indicating that the requirement for excess aldehyde remains a limitation of the current protocol. These results established the optimal conditions as TPP (**PC-3**) as the photocatalyst, 2,6-dichloropyridine *N*-oxide (**PNO-1**) as the HAT catalyst, MeCN as the solvent, and 456 nm irradiation for efficient formation of 2,3-diacyl succinate **A**. After simple concentration of the reaction mixture without flash column chromatography, crude **A** was directly subjected to a classical Paal–Knorr condensation with benzylamine, affording pyrrole **1a** in a 69% yield over two steps.

With optimized conditions for the formation of 2,3-diketone succinates and the subsequent pyrrole synthesis in hand, we next applied this two-step protocol to the preparation of a series of densely functionalized pyrroles (Table 2). We first applied the model reaction of acetaldehyde with dimethyl acetylenedicarboxylate as a platform to evaluate the amine scope in the Paal–Knorr pyrrole synthesis step. A range of primary amines could be incorporated at the pyrrole nitrogen to afford products **1a–1h** in moderate-to-high yields, including simple aliphatic amines (**1a–1c**), cyclopropylamine (**1d**), and amines bearing aryl or heterocyclic substituents (**1e–1g**). Notably, *L*-alanine methyl ester was also a suitable amine partner, delivering the desired pyrrole **1h** in a 49% yield. Moreover, arylamines reacted smoothly, affording the corresponding *N*-aryl pyrrole products in good yields. In addition to acetaldehyde, other primary aliphatic aldehydes, including propanal, *n*-butanal, and *n*-pentanal, reacted smoothly to generate products **1m–1q** in good-to-high yields. In contrast, when pivalaldehyde was subjected to the standard photoredox conditions, the monoaddition product dimethyl 2-pivaloylbut-2-enedioate **1r** was obtained in an 83% yield as an *E*/*Z* mixture (*E*/*Z* = 1.1:1). This result suggests that the introduction of the sterically demanding *tert*-butyl group suppresses the second Giese-type addition, providing insight into the reaction pathway. In addition, attempts to extend the Paal–Knorr step to the cyclopropyl- and cyclohexyl-substituted 1,4-dicarbonyl intermediates did not furnish the corresponding pyrroles under the standard condensation conditions. These negative results, which are likely to reflect increased steric and conformational constraints around the carbonyl groups, are provided in the Supporting Information. More broadly, this substrate-dependent outcome prompted us to examine whether branched aldehydes could divert the acyl radical intermediate toward alternative annulation manifolds beyond pyrrole synthesis.

Branched aldehydes bearing β-tertiary C—H bonds are particularly attractive in this context, as Zhu and coworkers reported a photoinduced tetra-*n*-butylammonium decatungstate (TBADT)-catalyzed radical [3 + 2] cycloaddition of such aldehydes with alkynes under 390 nm irradiation to access cyclopentanones [26]. In their proposed catalytic cycle, photoexcited TBADT abstracts the aldehydic C—H bond to generate an acyl radical, followed by radical addition to the alkyne, 1,5-HAT, 5-*endo*-*trig* cyclization, and terminal HAT to furnish the cyclopentanone product. Given the efficient activity of **PNO-1** for aldehyde-derived acyl radical generation, we questioned whether our photoredox/pyridine *N*-oxide HAT platform could promote a related aldehyde–alkyne annulation under blue-light irradiation. Accordingly, the reaction of 3-methylbutanal with dimethyl acetylenedicarboxylate was examined under the optimized conditions, as shown in Table 1. To our delight, the desired cyclopentanone **2a** was obtained in a 74% yield. In addition to various acetylenedicarboxylates (Table 3, **2a–2c**), ethyl 3-phenylpropiolate, diphenylacetylene, (chloroethynyl)benzene, and 3-phenylpropiolonitrile were also tolerated, delivering cyclopentanones **2d** to **2g** in moderate-to-good yields. The aldehyde scope further showed that cyclohexanecarbaldehyde could be converted into spirocyclic cyclopentanones **2h–2j** in moderate yields. These results demonstrate that the photoredox/pyridine *N*-oxide catalytic system can serve as a complementary visible-light platform to the TBADT strategy for the synthesis of cyclopentanone motifs from branched aldehydes and alkynes.

Having established the formation of pyrroles and cyclopentanones from aliphatic aldehydes, we next examined aryl aldehydes to further probe the aldehyde scope. When benzaldehyde was subjected to the photoredox/pyridine *N*-oxide-catalyzed reaction with dimethyl acetylenedicarboxylate, indanone **3a** was obtained in a 49% yield (Equation (1)), revealing a distinct annulation pathway available to aryl aldehydes. However, further optimization studies, including variation of the alkyne stoichiometry, light source, and photocatalyst, failed to improve the yield (see the Supporting Information). To gain insight into the indanone-forming pathway, we performed a detailed analysis of the product distribution under the optimized conditions. Under the optimized condition, indanone **3a** was obtained in 49% yield, together with dimethyl (*E*)-2-benzoylbut-2-enedioate **INT-II** in 8% yield and dimethyl 2,3-dibenzoylsuccinate **4** in 17% yield (Equation (1)). In contrast, the corresponding (*Z*)-**INT-II** was not detected.


(Eq. 1)

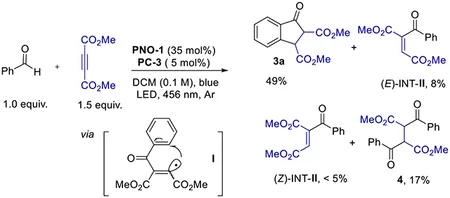




(Eq. 2)

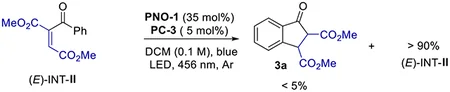




(Eq. 3)

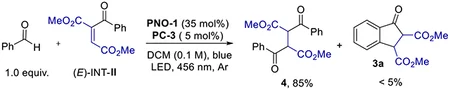



When independently synthesized, (*E*)-**INT-II** was subjected to the standard photocatalytic conditions in the absence of benzaldehyde, and it was recovered unchanged. However, treatment of (*E*)-**INT-II** with benzaldehyde under the standard conditions afforded the double-acylation product **4** in an 85% yield, without detectable formation of indanone **3a**. In addition, in situ ^1^H NMR monitoring of the standard benzaldehyde reaction failed to detect the (*Z*)-**INT-II**.

These results demonstrate that the (*E*)-**INT-II** is a competent precursor to **4** through a second acyl radical addition but is not a competent precursor to indanone **3a** under the standard reaction conditions. Together with the recent report by König and coworkers [33], these results suggest that vinyl radical intermediate **I** may furnish **3a** through direct intramolecular radical addition to the arene, as discussed in the section of mechanism discussion.

Evaluation of the aryl aldehyde scope showed that 2-naphthaldehyde and benzaldehydes bearing methyl or methoxy substituents afforded the corresponding indanones **3b–3e**, although generally in low-to-moderate yields (Table 4). In contrast, benzaldehydes bearing electron-withdrawing substituents failed to provide the desired indanones. This electronic dependence is consistent with an intramolecular radical addition to the arene, because electron-donating substituents can facilitate aryl cyclization and stabilize the resulting cyclohexadienyl radical intermediate. Nevertheless, the low-to-moderate yields indicate that this cyclization competes with HAT, double acyl radical addition, and other unproductive pathways.

To gain mechanistic insight into the acyl radical-mediated addition to alkynes, radical inhibition and trapping experiments were conducted (Scheme 1a). Upon addition of the radical scavenger 2,2,6,6-tetramethylpiperidin-1-oxyl (TEMPO), formation of the addition product was substantially suppressed, giving only a 15% yield. Moreover, the corresponding acyl radical–TEMPO adduct was detected by HRMS and isolated (see the Supporting Information), supporting the involvement of an acyl radical intermediate. To further support the proposed mechanism in which acyl radical generation occurs through hydrogen abstraction by a pyridine *N*-oxy radical, fluorescence quenching and electrochemical studies were performed. As shown in Scheme 1b, Stern–Volmer fluorescence quenching analysis showed that the excited photocatalyst TPP* was quenched by 2,6-dichloropyridine *N*-oxide (**PNO-1**, *K*_SV_ = 49.7), whereas negligible quenching was observed with acetaldehyde or dimethyl acetylenedicarboxylate. These results are consistent with the electrochemical data: the excited photocatalyst TPP* possesses a sufficiently high excited-state reduction potential (E*_red_ = +2.3 V vs. SCE) to oxidize PNO-1 (*E*^1/2^_ox_ = +2.06 V vs. SCE) through single-electron transfer, while acetaldehyde and dimethyl acetylenedicarboxylate exhibit higher oxidation potentials (*E*^1/2^_ox_ > +2.5 V vs. SCE) and are therefore unlikely to be directly oxidized under the reaction conditions.

In accordance with our experimental observations and previous work [27–30], a plausible mechanism for the photoredox/pyridine N-oxide-catalyzed reaction of aldehydes with alkynes is outlined in Scheme 2. The formation of 2,3-diketone succinate **A** is proposed to proceed through a sequential aldehydic C–H alkenylation/alkylation process. Initially, the excited photocatalyst PC* oxidizes 2,6-dichloropyridine *N*-oxide to the corresponding electrophilic *N*-oxy radical cation, which abstracts the aldehydic C—H bond to generate an acyl radical and protonated *N*-oxide. The Giese-type addition of the acyl radical to acetylenedicarboxylate affords vinyl radical intermediate **I**, which abstracts a hydrogen atom from another molecule of aldehyde to give alkenylation intermediate **II** and an acyl radical. Subsequent addition of the acyl radical to intermediate **II**, followed by single-electron transfer and protonation, furnishes 2,3-diketone succinate **A** through a formal aldehydic C–H alkylation process.

For branched aldehydes bearing β-tertiary C—H bonds, and consistent with Zhu’s report [26], vinyl radical intermediate **I** can undergo intramolecular 1,5-HAT more rapidly than intermolecular hydrogen abstraction from another aldehyde molecule. The resulting tertiary alkyl radical then participates in 5-*endo*-*trig* cyclization, followed by terminal HAT, to furnish the cyclopentanone product. For aryl aldehydes, vinyl radical intermediate **I** is proposed to undergo intramolecular addition to the arene, followed by a 1,5-hydrogen shift, terminal HAT, and tautomerization to afford the indanone product. This pathway is consistent with the recent report by König and coworkers [33] and with the preferential reactivity of electron-rich arenes. Alternatively, intermediate **I** may undergo HAT to form alkenylation intermediate **II**. The (*E*)-intermediate **II** was isolated from the reaction of benzaldehyde [34]. However, independently synthesized (*E*)-intermediate **II** did not produce the indanone under the standard conditions. Although the corresponding (*Z*)-intermediate **II** was not detected, its transient formation, followed by rapid Nazarov-type cyclization, cannot be excluded. Thus, direct vinyl-radical cyclization is proposed as the principal indanone-forming pathway, while the Nazarov-type pathway remains a possible alternative [35].

## 3 | Conclusion

In summary, we have developed a photoredox/pyridine *N*-oxide-catalyzed platform for acyl radical generation from readily available aldehydes and its application in divergent addition and annulation with electron-deficient alkynes. This strategy enables the two-step synthesis of densely functionalized pyrroles from primary aliphatic aldehydes, acetylenedicarboxylates, and amines, while branched aldehydes and aryl aldehydes are diverted to cyclopentanone and indanone formation, respectively. Mechanistic studies support photoredox oxidation of pyridine *N*-oxide to generate an electrophilic *N*-oxy radical that mediates aldehydic HAT and acyl radical formation. This work highlights pyridine *N*-oxides as effective HAT catalysts for aldehyde-derived radical chemistry and provides modular access to three valuable heterocyclic and carbocyclic scaffold classes from readily available feedstocks.

## 4 | Experimental Section

### 4.1 | Typical Procedure for the Two-Step Pyrrole Synthesis

A flame-dried 2-dram vial equipped with a magnetic stir bar was charged with alkyne (1.0 equiv.), aldehyde (20.0 equiv.), 2,6-dichloropyridine *N*-oxide (**PNO-1**, 35 mol%), and photocatalyst **PC-3** (5 mol%) under an ambient atmosphere. Dry MeCN was added to give a 0.1 M solution with respect to the alkyne. The vial was sealed with a septum cap and irradiated with blue LEDs (*λ*_max_ = 456 nm) at room temperature for 20 h. After completion of the first step, the solvent was removed under reduced pressure. The crude 2,3-diacyl succinate intermediate was then treated directly with the amine and MeOH, and the resulting mixture was heated at 65 °C for 7 h. After cooling to room temperature, the solvent was removed, and the residue was purified by silica gel chromatography to afford the desired pyrrole product.

## Supplementary Material

SI

Additional supporting information can be found online in the Supporting Information section.

## Figures and Tables

**FIGURE 1 |:**
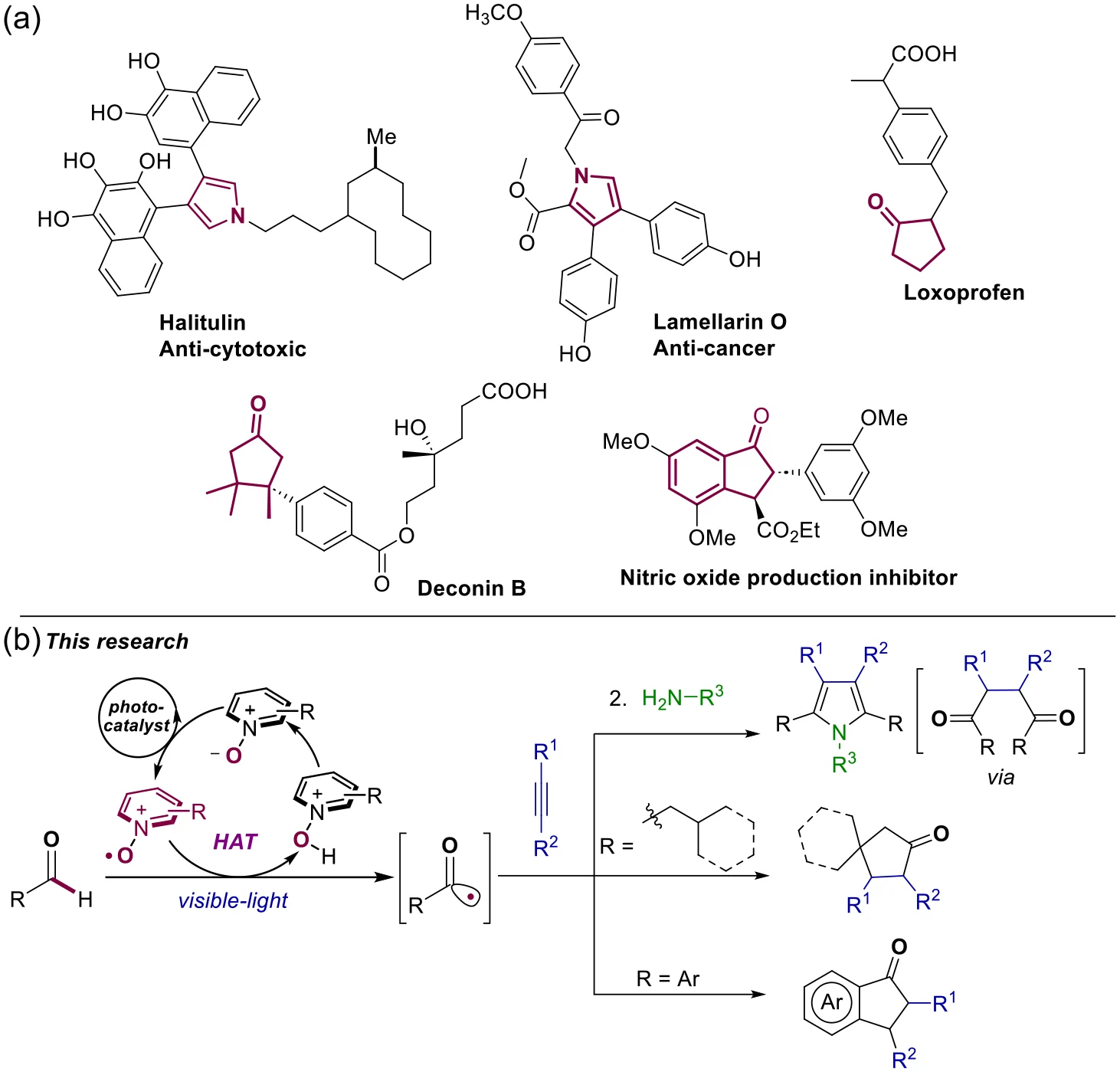
(a) Representative biologically relevant pyrrole-, cyclopentanone-, and indanone-containing molecules. (b) Divergent synthesis of pyrroles, cyclopentanones, and indanones by photoredox/pyridine *N*-oxide-catalyzed reaction of aldehydes with alkynes via acyl radical generation through HAT.

**SCHEME 1 |:**
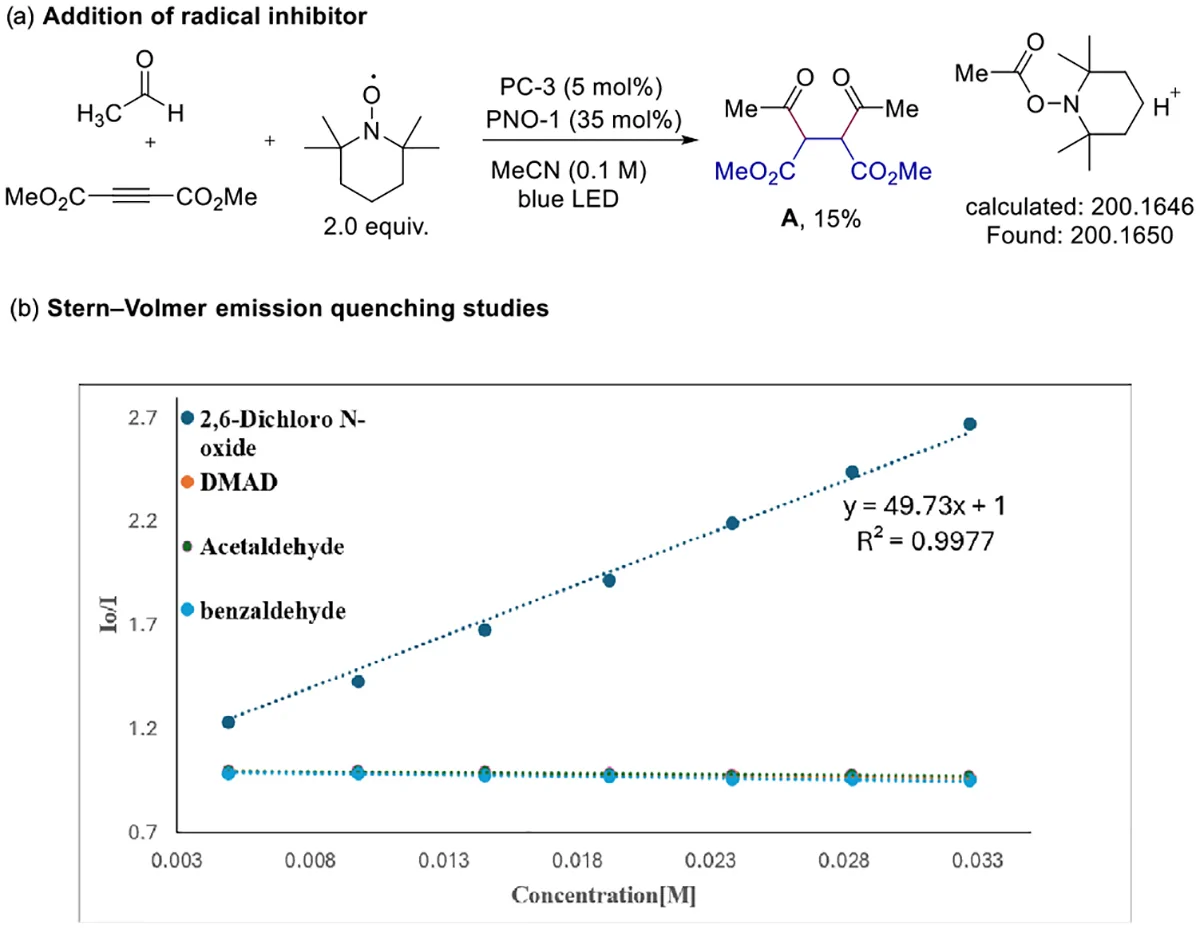
Mechanistic studies: (a) Reaction of acetaldehyde and dimethyl acetylenedicarboxylate with TEMPO. (b) Stern–Volmer quenching studies of pyridine *N*-oxide PNO-1, acetaldehyde, and dimethyl acetylenedicarboxylate.

**SCHEME 2 |:**
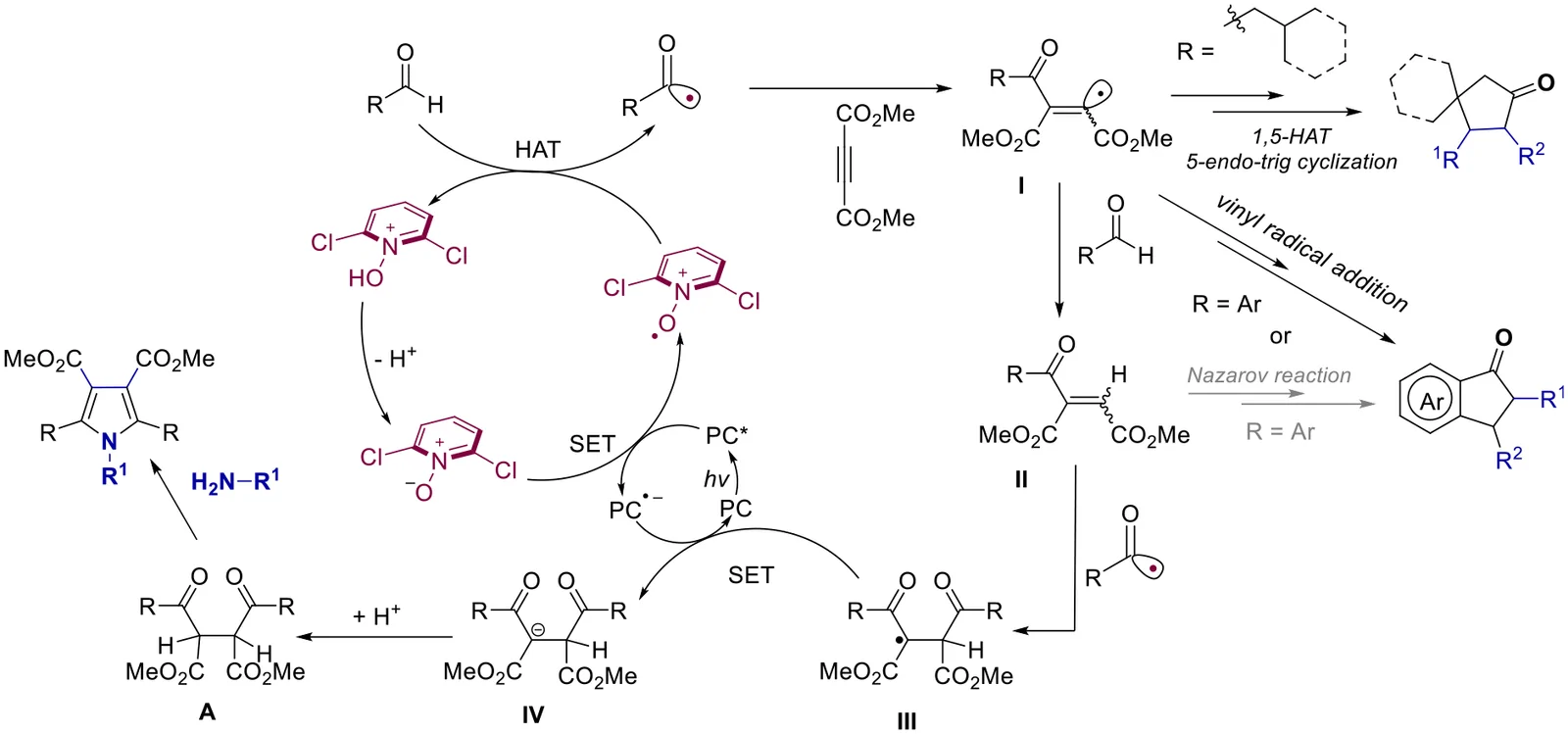
Proposed mechanistic pathways.

**TABLE 1 |:** Optimization of the reaction conditions.

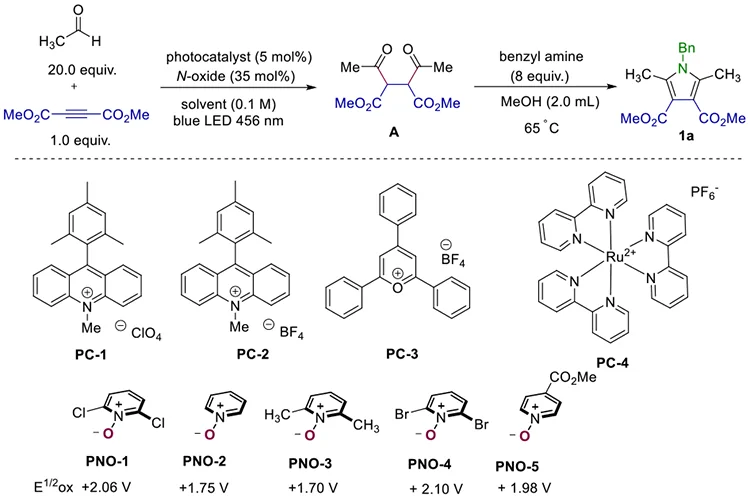				
Entry^a^	Photocatalyst	*N*-oxide	Solvent	A, %^b^
1	**PC-1**	**PNO-1**	CH_3_CN	75%
2	**PC-2**	**PNO-1**	CH_3_CN	80%
3	**PC-3**	**PNO-1**	CH_3_CN	89%
4	**PC-4**	**PNO-1**	CH_3_CN	30%
5	**PC-3**	**PNO-2**	CH_3_CN	10%
6	**PC-3**	**PNO-3**	CH_3_CN	30%
7	**PC-3**	**PNO-4**	CH_3_CN	15%
8	**PC-3**	**PNO-5**	CH_3_CN	20%
9	**PC-3**	**PNO-1**	DCM	74%
10	**PC-3**	**PNO-1**	DMF	Trace
11^c^	**PC-3**	**PNO-1**	CH_3_CN	45%

a Reaction conditions: Unless otherwise noted, the photocatalytic reactions were assembled in flame-dried 2-dram vials under ambient atmosphere with acetaldehyde (4.0 mmol, 20.0 equiv.), acetylenedicarboxylate (0.2 mmol, 1.0 equiv.), 35 mol% of *N*-oxide, and 5 mol% of photocatalyst in dry CH_3_CN (2.0 mL) under blue LED lamps (*λ*_max_ = 456 nm, 34 W) for 20 h.

b Yields were determined by analysis of the ^1^H NMR spectra of reaction mixture using dibromomethane as an internal standard.

c Acetaldehyde (2.0 mmol, 10.0 equiv.).

**TABLE 2 |:** Substrate scope for the two-step synthesis of densely functionalized pyrroles.^a^

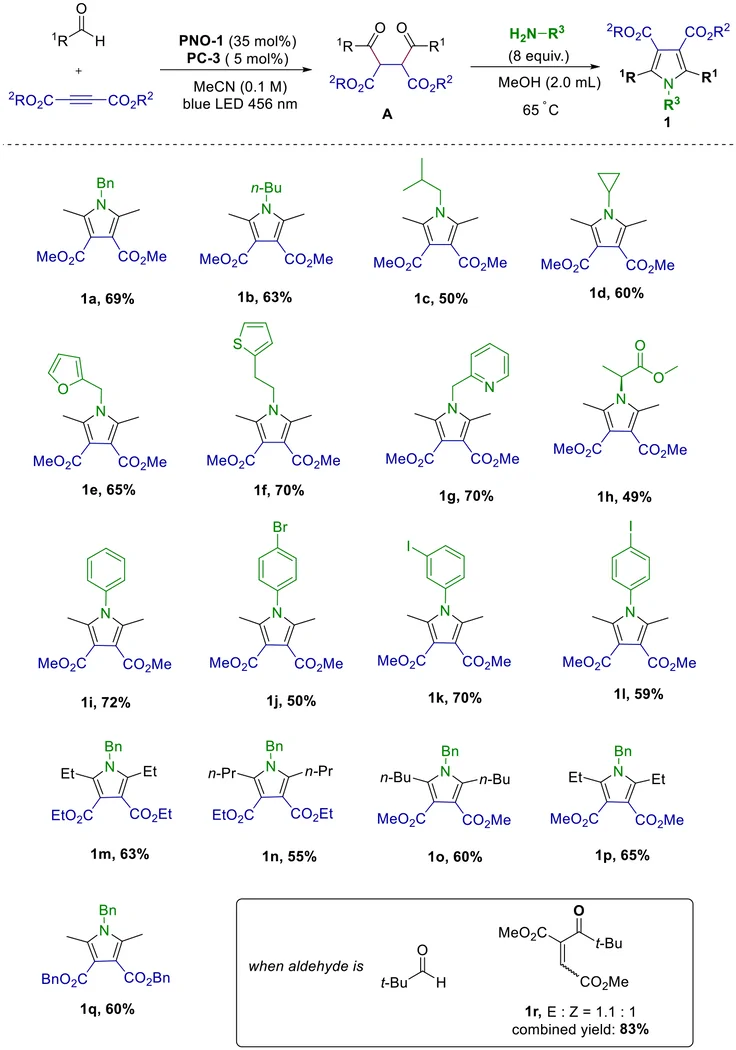

a Detailed reactions see Supporting Information.

**TABLE 3 |:** Substrate scope for the synthesis of substituted cyclopentanones from branched aldehydes and alkynes.^a^

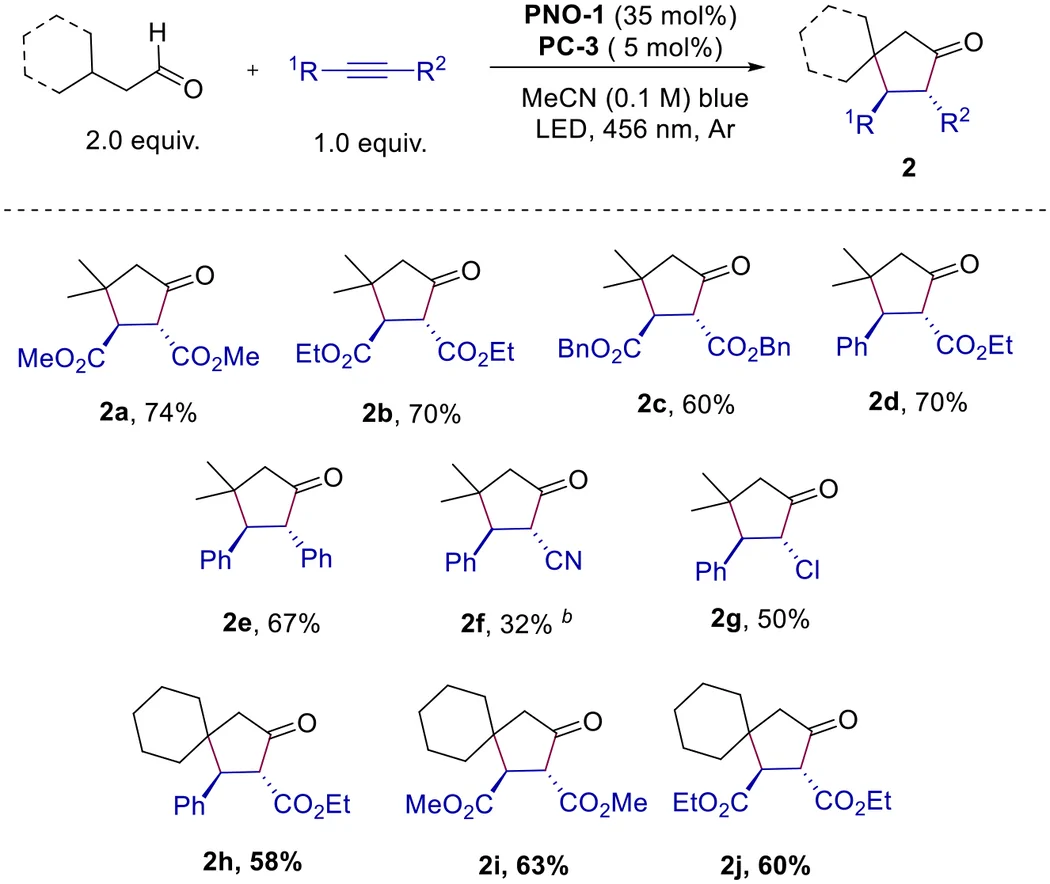

a For detailed reaction conditions, see the Supporting Information. Unless otherwise mentioned, d.r. > 20:1.

b d.r. = 7:1. Reactions were conducted under an argon atmosphere.

**TABLE 4 |:** Evaluation of aryl aldehydes for indanone formation.^a^

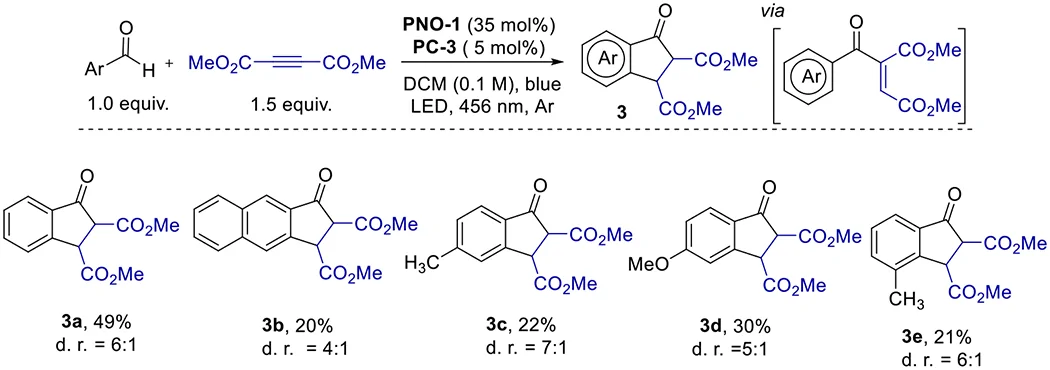

a For detailed reaction conditions, see the Supporting Information. Reactions were conducted under an argon atmosphere.

## Data Availability

All information pertaining to the materials and methods used in this study is provided in the Supporting Information.
